# Socialization, Education, and Learning for the Internet (SELFI): A Pilot RCT of a Social Media Skills Group Program for Autistic Adults

**DOI:** 10.1007/s10803-023-06100-9

**Published:** 2023-08-16

**Authors:** Anthony Osuna, Katie Sabini, Eryca Yamane, Jaqueline Flores, Naomi Pierce, Jocelyn Lemus-Valle, Ty Vernon

**Affiliations:** 1 Koegel Autism Center, Department of Counseling, Clinical and School Psychology, University of California Santa Barbara, Santa Barbara, CA 93117 USA; 2 Center for Child Health, Behavior and Development, Seattle Children’s Research Institute, 1900 9th Ave, Seattle, WA 98101 USA; 3 Department of Psychiatry & Behavioral Sciences, University of Washington, 1959 NE Pacific Street, Box 356560, Seattle, WA 98195-6560 USA

**Keywords:** Autism, Autistic Adults, Social Media, Social Skills, Program Development

## Abstract

Many autistic adults report preference for computer-mediated communication and social media use. Despite many benefits to online socialization, there are many challenges including anxiety and cyber-victimization. To date, support is limited related to helping autistic adults with safe and effective internet use. The purpose of this study was to evaluate the feasibility, acceptability, and preliminary efficacy of the novel SELFI program. This pilot study utilized a randomized controlled trial design. A total of 25 autistic adults enrolled in the study and were randomized to the nine-week SELFI program or a waitlist control condition. Feasibility assessed enrollment, attrition, and fidelity of delivery. Acceptability examined attendance and feedback from participants and peer mentors. Efficacy evaluated change in Facebook activity, social media utility/anxiety, and individualized goals. Regarding feasibility, the recruitment goal was met within one month, there was limited attrition, and therapists delivered the program with high fidelity. Participants attended a majority of scheduled sessions and feedback from participants reflected high levels of agreement with several facets of the program. Compared to the control group, more participants assigned to the SELFI condition were perceived by autistic and non-autistic raters as having improved Facebook activity. SELFI participants also reported reduced difficulty meeting their individualized goal. Findings support the piloted SELFI program as feasible and acceptable with signals of preliminary efficacy. This study establishes an exciting foundation regarding an innovative social media skills program, however more research is necessary.

Social networking sites (SNSs) are web-based platforms that allow users to engage with others in real time or asynchronously using computer-mediated communication for the purpose of generating and maintaining social connections (Bayer et al., 2020). A subclass of SNSs are considered *social media* applications, which are applications that have an account profile, social network, and content stream, to support self-presentation, social mobilization, and social connection (Ellison & Boyd, 2013) In the United States, approximately 72% of young adults are active on social media (Auxier & Anderson, 2021). Autism spectrum disorder (ASD) is characterized by challenges in social communication and the presence of restricted and repetitive interests and behaviors (American Psychiatric Association, 2013). In congruence with published suggestions for autism researchers to avoid ableist language (Bottema-Beutel et al., 2021), identity-first language (“autistic adult”) is used within this study as opposed to person-first (“adult with autism”) or mixed-language. Autistic people may have challenges with face-to-face social interactions as well as broader difficulties around core social skills competencies (Silveira-Zaldivara et al., 2021), including interpreting the social intent of others and knowing which skills to utilize within specific contexts (Hanley et al., 2015). Research on social media use is scarce, however recent studies estimate that approximately 79–84% of autistic adults may use SNSs (Mazurek, 2013; Ward et al., 2018). Social networking sites have been described as a preferable medium of social communication for some autistic people due, in part, to online interactions not relying on the same non-verbal cues (e.g. eye contact, gestures) that are present within face-to-face interaction (Stendal & Balandin, 2015). Social media offers more visual cues than in-person socialization and greater degrees of asynchrony (Nesi et al., 2018), which may provide autistic adults with increased comprehension and control over interactions (Gillespie-Lynch et al., 2014). Recognizing that some autistic people may find in-person socialization anxiety provoking or over-stimulating (Joseph et al., 2008), social media offers a potentially more comfortable milieu for social engagement.

Social media grants access to valuable tools and opportunities for maintaining existing relationships and seeking new friendships. Autistic adults are more likely to be socially isolated (Orsmond et al., 2013) and many cite social connection as their primary reason for using social networking sites (Mazurek, 2013). Autistic adults who are active on social media report having closer friendships, improved well-being, and are happier than those who do not socialize online (Mazurek, 2013; Ward et al., 2018). Social media offers increased opportunities to meet others with focused interests and participate in the neurodiversity movement (Wang et al., 2020; Kapp et al., 2013). While there are many benefits to increased socialization opportunities over the internet, social media use has been linked to several negative consequences regarding mental health. Amongst the general population of adults, social media has been associated with increased symptoms of anxiety, depression, dissatisfaction with one’s body, and lowered self-esteem (Sherlock & Wagstaff, 2019). Additionally, internet use can become addicting and maladaptive, which may lead to negative consequences on one’s mood and other important areas of life (Andreassen et al., 2017). Although online socialization may offer autistic adults unique benefits, the challenges associated with autism may make it difficult to safely and appropriately navigate social media. Higher scores on the Autism Quotient (Baron-Cohen et al., 2001), a measure of behaviors associated with autism, have been associated with fewer online interactions with friends, associations with social media groups, hours on Instagram, and posts to Instagram per week (Suzuki et al., 2021). Autistic adults report specific social media challenges related to increased anxiety, harassment, drama, privacy concerns, cyber-victimization and knowing who to trust online (Wang et al., 2020; Triantafyllopoulou et al., 2021; Gwynette et al., 2018; Burke et al., 2010).

Safe and effective online interaction requires a distinct set of social skills. Many of these strategies relate to social skills used within face-to-face interactions, however some are unique to the internet and may require experience or explicit training to refine. For example, navigation of computer-mediated communication depends on use of complex emojis, which may lead to misunderstanding if misinterpreted (Nesi et al., 2018). Successful navigation of online relationships requires a nuanced understanding of social media tools (e.g. likes, comments, posts) and etiquette, including when to send friend requests and what type content to post within a specific environment (Pham et al., 2019). *Social competence* involves understanding which skills and strategies are likely to elicit a desirable response within the context of a particular interaction (Silveira-Zaldivara et al., 2021). Considering that social skills are environmentally specific and contextually reinforced (Little et al., 2017), effective social media use likely depends on the development of online-specific social competence.

Many autistic people experience difficulties with developing social competence. Although challenges with social competence can be attributed to one’s inherent neurodiversity, it is also understood that those with social challenges experience more difficulties due to the transactional nature of social development (Jones & Klin, 2009). This is, increased social experiences help develop social competence by providing feedback and opportunities to refine skills. Targeted interventions that address social skills through didactics (Stichter et al., 2010), group discussion (e.g., MacKay et al., 2007), rehearsal opportunities (Minihan et al., 2011), role-plays, and modeling (Tse et al., 2007), have been demonstrated to be effective at improving social competence among autistic individuals (Miller et al., 2014). These strategies have led to social competence improvement in areas related to responding to others (McMahon et al., 2013), initiating conversations (Mitchell et al., 2010), and knowledge regarding how to engage in certain social situations (Vernon et al., 2016). While social skills programs targeting face-to-face interactions have been shown to improve outcomes among autistic individuals (Spain & Blainey, 2015), little research has explored social support specifically designed for online socialization. While some face-to-face social skills programs, such as PEERS (Laugeson et al., 2015) and START (Vernon et al., 2018) address digital social communication (texting, video chatting, emailing, etc.) as a single lesson within their curriculum, these limited supports are likely insufficient for developing the nuanced and idiosyncratic social competency required for online social interaction.

The Socialization, Education, and Learning for the Internet (SELFI) program is a novel social media skills program designed to help autistic adults with safe and effective internet use. The SELFI program focuses on increasing knowledge related to online socialization, social opportunities, and use of prosocial behavior. Development of the SELFI program was guided by theories of social competence (Silveira-Zaldivara et al., 2021), evidence-based practices (Reichow & Volkmar, 2010), and identification of skills that are likely to elicit desirable social consequences. The curriculum includes nine sessions related to having an appropriate online profile, introducing yourself, responding to requests for personal information, associating with groups, responding to and refraining from cyberbullying, disagreeing with others, and understanding your audience (Morgan et al., 2016). An emphasis is placed on highlighting that use of skills requires the ability to flexibly vary online behavior depending on your relationship with the other individual (Bryant & Marmo, 2012). The curriculum is delivered using evidence-based practices, such as didactic lessons and experiential learning (Kolb, 2014), which are used within face-to-face social skills programs (Vernon et al., 2018; Laugeson et al., 2012; Webb et al., 2004; White et al., 2010). SELFI focuses on making abstract concepts more concrete, offering varied learning opportunities, fostering partnerships, choosing relevant goals, teaching skills sequentially, and providing opportunities for skill generalization through practice (Krasny et al., 2003). Regarding structure, the SELFI program follows the Social Tools and Rules for Rules for Teens (START) program (Vernon et al., 2018) as a model.

The goal of SELFI is to improve social competence in order to increase online social engagement, which is hypothesized to improve mental health outcomes. The hypothesized mechanism of change for SELFI is based on previous findings of autistic social media use. Among autistic adults, loneliness has been associated with challenges with depression, anxiety, and quality of life (Mazurek, 2014). More and better quality friendships have been associated with lower levels of loneliness (Mazurek, 2014). Autistic adults who use social media report more close friends and closer relationships (Mazurek, 2013). The association between autistic social media use and friendship quality have been reported to be moderated by anxiety levels (van Schalkwyk et al., 2017). Therefore, it is hypothesized that instruction in the SELFI program will lead to improved social competence in social media skills [instruction → knowledge acquisition (proximal mechanism)] resulting in increased social engagement [knowledge acquisition → implementation of social skills (short-term distal outcome)]. This, in turn, should decrease loneliness [skill implementation → decreased loneliness (short-term distal outcome)]. A decrease in loneliness may have long-term outcomes including reduced anxiety and depression and increased quality of life (long-term distal outcomes).

A prototype of the SELFI program was pilot tested with six autistic adults to explore initial feasibility and acceptability using a single case experimental design (Osuna et al., 2023). The purpose of this study was to assess the key feasibility characteristics of a planned research methodology prior to engaging in a larger study (Moore et al., 2011). Of the six that enrolled, each participant completed the study and attended all scheduled SELFI sessions. The program was delivered with high fidelity (85.1%) and participants endorsed high levels of satisfaction with the program. Each participant demonstrated individual increases in Facebook likes/reactions, comments, and posts. These behaviors were chosen due to being socially relevant indicators of active and passive online engagement that impact well-being (Verduyn et al., 2017). Active social media usage refers to actions that facilitate direct exchanges with others while passive usage refers to the monitoring of other people’s lives without directly engaging (Burke et al., 2010). Programmatic feedback included making the content more advanced, working on individualized goals, helping participants remember session content, and adding content related to online romance and dating. This study was an important first step for formulating a novel intervention technique (Smith et al., 2007), however further program development is necessary.

The current study sought to pilot a refined version of the SELFI program modified considering feedback from the initial prototype trial. In addition to introducing new features to the intervention, the structure of the SELFI program was changed from a 1:1 format delivered face-to-face to group format delivered over telehealth. In response to face-to-face limitations due to the COVID-19 pandemic, this study utilized a fully remote research and clinical protocol. The overarching aim of this research was to produce a group program to be provided over telehealth. Adaptations to the SELFI curriculum included expanding curriculum to be more advanced (including the addition of a session related to online romance) and adding evidence-based strategies, such as visual supports to support content retention (Shane et al., 2012) and collaborative goal setting to support individualized needs (Costa et al., 2017). Use of peer mentors, another evidence-based strategy (Watkins et al., 2015), was added to the intervention to provide participants with more personalized support within the revised group format. Considering the untested nature of the revised SELFI program, the primary focus of this study was to assess key feasibility and acceptability characteristics of the research protocol and identify potential concerns and necessary modifications prior to more rigorous testing (Thabane et al., 2010; Czajkowski et al., 2015). Within this scope, the primary aims of this study were to assess the preliminary feasibility and acceptability of the novel SELFI program. A secondary aim was to explore signals of efficacy on outcomes relevant to social media, using Facebook as a proxy for online social engagement. For this study, feasibility examined evidence that the study protocols could be adequately completed, considering study enrollment, program attendance, and therapist fidelity. Acceptability explored evidence that the study was appropriate, reflected in attrition and feedback from participants and peer mentors. Regarding program efficacy, it was hypothesized that more participants in the experimental condition would demonstrate increased frequencies of Facebook likes/reactions, comments, and posts. Additionally, more participants in the experimental condition would be perceived as having *improved* social engagement on Facebook, as rated by autistic and non-autistic raters. It was also hypothesized that the experimental condition would demonstrate greater differences in social media utility and social media anxiety and that participants would report reduced difficulty regarding a personally relevant social media goal after the trial.

## Methods

### Design

A pilot randomized control trial (RCT) with a waitlist-control condition was used to pilot the group SELFI program. Participants were randomly assigned to the SELFI program or a nine-week waitlist condition. Stratified random sampling was used due to its ability to produce balanced gender samples across groups (Kim & Shin, 2014). Outcome measures were collected at baseline and week 9. Control participants were offered the opportunity to enroll in the SELFI program after completion of the RCT. Feasibility and acceptability data were gathered from all participants who enrolled in the SELFI program. Program feedback was gathered from all participants and peer mentors. Efficacy data was only collected within the nine-week trial. This study was approved by the study site Human Subjects Committee and all participants provided informed consent prior to inclusion in the study.

### Participants

A total of 25 autistic adults (*M age* = 24.6 years; SD = 5.88) without intellectual disability enrolled in the study. Of these participants, 18 identified as male and 7 identified as female. Inclusion criteria included a diagnosis of autism spectrum disorder, aged 18–40 years, English speaking, residence within the United States, an active Facebook account, and a verbal composite score ≥ 70 using the Kaufman Brief Intelligence Test, Second Edition (KBIT-2; Kaufman, 2004). An age range between 18 and 40 years was used to control for generational differences in social media preferences. Limiting participation to those who speak English and live within the United States was due to resource limitations. An active Facebook account was necessary in order to standardize data collection related to social media engagement. A verbal composite score ≥ 70 was used as a cutoff due to the verbal demands of the piloted program and in accordance with Krasny et al. (2003) who recommended that social skills programs group participants by language ability. The mean verbal composite score of participants was 88.2 years (SD = 10.1), which falls in the low average range. Clinical autism diagnoses were provided by community providers and confirmed by the research team. The Social Responsiveness Scale, Second Edition (SRS-2; Constantino & Gruber, 2012) was used to describe autism characteristics at baseline. The mean SRS-2 t-score of all participants was 63.3 (SD = 9.1; 91st percentile), which falls in the high average range and reflective of elevated autism characteristics . The average length of social media experience was 7.0 years (SD = 5.4). Participant recruitment was entirely digital and consisted of circulating a recruitment flier and eligibility screener to relevant organizations and postings to social media. Each participant received a $10 gift card as incentive for completing baseline and week 9 surveys for a total of $20. See Table 1 for a summary of participant characteristics.

**Table 1:** Participant demographic characteristics

Variable	Treatment (n = 12)				Waitlist (n = 13)			
	*n*	%	*Mean*	*(SD)*	*n*	%	*Mean*	*(SD)*
Female	3	25%			4	31%		
Male	9	75%			9	69%		
Age (years)			25.3	6.9			23.9	4.9
KBIT-2 Verbal Composite score			88.0	12.0			88.3	8.4
SRS-2 Total t-score	12		60.6	10.6	13		65.8	6.7
**Ethnicity**								
White	6	50%			5	38.5%		
African American/ Black	0				1	7.7%		
Asian American/ Asian	1	8.3%			4	30.7%		
Hispanic/ Latinx	1	8.3%			3	23%		
Multiracial	2	16.7%			0			
Other	2	16.7%			0			
**Education**								
Graduate Degree	1	8.3%			0			
College Degree	1	8.3%			1	7.7%		
Some College	4	33.3%			6	46.1%		
High School/GED	6	50%			6	46.1%		
**Income**								
Less than $25K	7	58.3%			8	61.5%		
$25K-$49K	2	16.7%			3	23%		
$75K-$99K	1	7.7%			0			
$100K-$149K	1	7.7%			2	15.4%		
$150K+	1	7.7%			0			
**Relationship Status**								
Single	10	83.3%			12	92.3%		
Relationship	1	8.3%			1	7.7%		
Married	1	8.3%			0			

Note: There were no significant differences between conditions for age, KBIT-2 verbal composite scores, and SRS-2 scores

### Measures

#### Participant Characterization

##### Recruitment Screener

This digital survey was used to gather data related to study recruitment and to screen for initial inclusion criteria (age, location, use of Facebook, autism diagnosis).

##### Demographic Data Form

This form was provided to enrolled participants to gather data related to gender, age, ethnicity, education attainment, income, relationship status, and social media experience.

##### Kaufman Brief Intelligence Test, Second Edition (KBIT-2, Kaufman, 2004)

The KBIT-2 is a brief cognitive screening tool that takes about 30 min to administer. Due to remote administration and a focus on assessing verbal ability, only the verbal composite subtests were administered. The verbal composite score of the KBIT-2 consists of two subtests (Verbal Knowledge and Riddles) and has a mean of 100 and standard deviation of 15. Psychometric properties place internal reliability for the Verbal scale at α = 0.91 and test-retest reliability ranging from 0.88 to 0.89.

##### The Social Responsiveness Scale, Second Edition (SRS-2; Constantino & Gruber, 2012)

The SRS-2 is a 65-item rating scale that measures the severity of autism spectrum characteristics as they occur in natural settings. Participants completed the adult self-report form, which takes approximately 15 min to complete. Psychometric properties on the SRS-2 have high internal consistency (α = 0.95) and inter-rater agreement coefficients ranging from 0.72 to 0.82.

#### Outcome Measures

##### Treatment Fidelity Checklist

All sessions were recorded and 20% (eight sessions) were reviewed by research assistants for treatment fidelity. Checklists were created for each core session of the SELFI program. Group facilitators were rated on adherence to specific events that should have occurred during each treatment session using a scale 0–1 (0 = goal was not achieved; 1 = goal was achieved). Fidelity training was led by the principal investigator and included a thorough review of the curriculum. Practice fidelity took place over two weeks and included reviewing 20% of recorded videos and reaching 100% interrater reliability with the primary investigator. Discrepancies in training were resolved before raters scored a separate sample of eight randomly selected sessions.

##### Participant Feedback Survey

An anonymous online feedback survey was administered to participants after completion of the SELFI program. Survey questions asked participants to rate their experience on a 5-point scale (1 = strongly disagree to 5 = strongly agree). Likert scale questions related to whether the program was satisfactory, enjoyable, and helped with areas of online socialization, including: increasing confidence online, improving online safety, maintaining relationships, making new friends, and navigation of online romance. Open-ended questions related to the acceptability of program materials and procedures, including most helpful aspects, favorite and least favorite components, suggestions, and recommendations for future programming.

##### Peer Mentor Feedback Survey

An anonymous survey online was provided to peer mentors after completion of the trial. Questions asked mentors to rate their enjoyment and perceived benefit of mentorship on a 5-point scale (1 = strongly disagree to 5 = strongly agree). Mentors also provided open-ended feedback regarding likes, dislikes, strategies used, and suggestions for future programming.

##### Social Media Experience Scale (SMES; van Schalkwyk et al., 2017)

The SMES is an 11-item scale designed to measure feelings of anxiety experienced in the course of using or contemplating social media, and the ways in which it was considered useful. Items were derived from a prior qualitative study of social media experiences in a clinical population (van Schalkwyk et al., 2014). The measure was designed to have two subscales, SMES-Anxiety and SMES-Utility. The SMES-Utility subscale measures active engagement on social media, rather than time spent online which could also represent passive observation of social media platforms. SMES-anxiety measures stress related to social media use. An exploratory factor analysis (EFA) with 56 autistic and non-autistic adolescents revealed two factors with eigenvalues greater than 1 (van Schalkwyk et al., 2017). One item (“I worry about my replies to people”) was inconsistent with its intended scale and omitted from analysis, resulting in a 10-item scale. Total score for the SMES-Anxiety factor was highly correlated with total score on the Multidimensional Anxiety Scale for Children 2nd edition (MASC-2; March et al., 1997) child in both autistic (r = .45, p < .01) and non-autistic samples (r = .44, p < 01).

##### Facebook “Download Your Information” (DYI)

Participants requested a downloadable file containing a chronological log of their Facebook activity during a designated timeframe using a feature native to the website called Download Your Information (DYI; https://www.facebook.com/dyi). This file contained data related to each participant’s Facebook likes/reactions, comments, and posts. Facebook was chosen as the platform of interest due to being the most popular amongst autistic adults (Ward et al., 2018) and the only major social media website to offer a standardized process for accessing a chronological history of online social behavior. Facebook activity from the seven days before and after the trial were evaluated as a proxy for recent online social engagement.

##### Individualized Social Media Goal Difficulty Rating

Participants in the experimental condition identified one individualized social media goal. To support goal selection, peer mentors provided participants with a list of common social media domains and helped them select one that they wanted to target within the program. Participants were allowed to choose a goal that was not listed. Goal operationalization was informal and not tracked. Selected goals related to feeling confident online, maintaining relationships using social media, understanding boundaries online, using social media to strengthen offline friendships, and navigating romance. Participants rated their level of difficulty meeting this goal (1 = very easy to 7 = very difficult) at baseline and week 9.

### Procedure

Interested individuals completed an online recruitment screener to assess initial eligibility criteria. Those who passed the screener were contacted by a member of the research team to schedule a virtual intake appointment (via Zoom) to gather informed consent and assess full eligibility using the KBIT-2. Individuals who met eligibility criteria were enrolled in the study and completed baseline assessments. Those who were not eligible to participate were provided alternative resources for social support. Enrolled participants submitted a file containing their previous seven days of Facebook activity using the DYI feature. Only data pertaining to Facebook likes/reactions, comments, and posts were requested, while more personal information such as direct/private messages were omitted. Participants downloaded this file while logged into their own account and submitted it to the research team via secure cloud service. Participants were assigned using stratified random sampling. Male and female participants were randomized separately to control the gender distribution across conditions. Twelve participants were assigned to the experimental condition and 13 to the control condition. Participants randomized to the experimental condition were assigned to one of three SELFI groups, which met at the same time each week to maintain consistency. Those assigned to the control condition were placed on a nine-week waitlist. Upon initiating the SELFI program, participants worked with their assigned peer mentor to identify an individually relevant social media goal and rate their level of difficulty. A list of potential goals was provided to support goal identification. All participants completed outcome measures and submitted another seven day sample of their Facebook activity after completion of the trial at week 9. Participants assigned to the control condition were offered the opportunity to enroll in the SELFI program after completion of the RCT at week 9. Data from waitlist SELFI groups were collected regarding the feasibility and acceptability of the program.

#### SELFI Program

The piloted SELFI program was delivered in group format over teleconference (via Zoom). Participants were encouraged to keep their cameras on during sessions but were allowed to turn them off when preferred. Attendance was recorded at the start of each session. Weekly sessions were 60 min and followed an established curriculum, progressing from basic to more advanced skills (see Table 2 for an overview of the SELFI curriculum). Each SELFI group consisted of four participants, two non-autistic peer mentors, and one group facilitator (graduate student in psychology). Each peer mentor was assigned to support two participants. All session material, content, and procedures were outlined using presentation slides (e.g. PowerPoint) and displayed to participants using Zoom’s screenshare feature. Session format consisted of a check-in (10 min), group video example (10 min), topic discussion (20 min), practice activity (15 min), and check-out (5 min). Check-ins were conducted by peer mentors in private breakout rooms and used to summarize the previous week’s material, answer related follow-up questions, and to prime the participant for the upcoming discussion. After check-in, participants returned to the main room for the video and topic discussion. Video examples were pulled from YouTube, vetted for appropriate content, and broadcasted to the group as an opportunity to introduce session material using footage from popular sources (e.g. Ellen Show, Buzzfeed). Topic discussions were centered around the weekly social media subject and followed a predetermined didactic curriculum that included specific forms of online safety that are important for all internet users and to which autistic individuals may be particularly vulnerable. For example, week 5’s lesson “Internet Safety” reviewed strategies for identifying and responding to deceptive and harmful online behaviors, such as scams, internet trolls (deliberately trying to offend, cause trouble, or attack others) and catfishing (when someone pretends to be someone else online). Within group discussion, participants and peer mentors were encouraged to share and solicit relevant personal examples. Target skills were presented as “rules of thumb” and provided explicit and trackable guidelines regarding the frequency and intensity of certain behavior (e.g. try to like 5–10 posts per day). An emphasis was made to differentiate how use of rules depend on one’s relationship with the other user. Practice activities also took place within breakout rooms and utilized experiential learning, modeling, rehearsal, and support from peer mentors. These activities involved interactive worksheets, practicing using their own social media accounts, and receiving in-vivo feedback while screen sharing. During the five-minute checkout, peer mentors summarized session content, solicited remaining questions, and encouraged skill generalization between sessions in the form of “homework.” Homework assignments were predetermined and consistent across participants, however completion was not tracked. After each session, each participant was emailed a handout which summarized the key points from the lesson.

**Table 2:** SELFI curriculum

Week	Topic	Description	Example Rules
1	Establishing an Online Presence	Tips for setting up an account and profile page	Share information about yourself on your profile, Be honest and positive
2	Being a Good Internet Friend	Responding thoughtfully, avoiding arguments	Don’t post anything that will hurt someone’s image, career, or relationships
3	Types of Online Relationships	How to navigate different types of relationships online (friends vs. acquaintances)	Use privacy settings to control how much information to share with certain people
4	Responding to Others	How to reciprocate online social communication	Take your time but be punctual, Reply to your friends, be careful engaging with strangers
5	Internet Safety	How to prevent and respond to online challenges	Block and report harassing and threatening interactions
6	Relationship Maintenance	How to keep in touch with people known in-person	Like your friend’s posts, Leave them a happy birthday message
7	Making New Connections Online	How to use social media to develop new relationships	Review someone’s social media account to learn more about them
8	Romance and Online Dating	How to seek romantic relationships online	Use dating websites and apps, discuss romantic intent

#### Facebook Improvement Ratings

To assess changes in online social competency, Facebook behavior was rated for perceived level of improvement. To do this, an online survey was created with each page containing screenshot photos of each participant’s Facebook likes/reactions, comments, and posts at baseline and week 9. To control for participants who had high frequencies of Facebook activity, only the most recent 10 likes, 10 comments, and 10 posts were displayed since the focus was on the level of improvement, rather than the quantity of specific actions. Each page of the survey presented seven days of Facebook likes/reactions, comments, and posts from the same participant at baseline and week 9. Responders were instructed to spend at least 1 min reviewing the entirety of the presented data and asked to form an impression considering post frequency and “the impact that you believe their activity would have on their social relationships.” At the bottom of each page, responders were prompted to provide a rating using a seven-point Likert scale (1 = strongly disagree, 4 = neutral, 7 = strongly agree) in response to the prompt “this person’s Facebook activity improved.” The order in which each participant was presented was counterbalanced.

To obtain a diverse range of perspectives, 12 similarly aged (M = 22.1 years; SD = 3.3) research assistants were recruited to provide improvement ratings. These research assistants were recruited through a university autism center. Raters were selected if they reported having an active Facebook account and experience using social media. An equal ratio (three each) of autistic males, autistic females, non-autistic males, and non-autistic females was chosen so that neurodiverse and gendered perspectives were weighted equally. Recruitment of non-binary raters was attempted but unsuccessful. Raters were unaffiliated with the study’s previous protocols and unaware of participant condition. The mean improvement score of each participant was calculated using the ratings of all 12 research assistants.

### Statistical Analyses

All statistical analyses were conducted using SPSS 28.0. A preliminary analysis of the primary aims calculated descriptive statistics for feasibility and acceptability outcomes. Analysis of feasibility and acceptability variables included data from all participants who enrolled in the SELFI program. Program feasibility considered enrollment rates, attendance, and fidelity of delivery. Enrollment calculated the number of individuals who were screened and randomized. The experimental and control groups were compared across demographic variables for equivalency. Attendance was computed by dividing the total number of attended sessions by the total number of possible sessions. Fidelity was analyzed by dividing the number of successfully delivered program components by the number of specific events that should have occurred. We set a goal of 80% fidelity to the session content to support the claim that the manual can be delivered in a uniform manner (Johnson et al., 2007). Program acceptability evaluated study attrition and survey feedback from participants and peer mentors. Attrition calculated the percentage of participants who dropped from the study. Analysis of feedback surveys calculated the average response rating for Likert scale questions and summarized responses to open-ended questions.

Outcome data were analyzed after completion of the RCT at week 9. A preliminary analysis of outcome measures assessed for outliers, normality, and missing data. Total scores were calculated for the SMES-Utility and -Anxiety subscales and converted to difference scores (DS; Week 9 - Baseline). For SMES-Utility, positive DS indicated increased social media utility and negative DS reflects reduced utility. For SMES-Anxiety, negative DS indicated reduced social media anxiety and positive DS represents increased levels. Preliminary analysis revealed one extreme outlier for the SMES, which was removed from subsequent analysis. A Hotelling’s T^2^ (one-way MANOVA) was run to determine the effect of condition on DS for the two SMES subscales. The distribution of frequencies for Facebook-related variables violated assumptions of normality. Therefore, an emphasis was placed on detecting minimal clinically relevant differences in activity between baseline and week nine. For each Facebook variable (likes/reactions, comments, posts, and improvement), participants who demonstrated an improved score were coded as “responders’’ and those who did not were coded as “non-responders.” Due to small sample sizes, traditional Pearson χ2 tests were not appropriate and so the proportion of positive responders between groups was compared using Fisher’s exact tests. Proportions refers to the ratio of responders in each group who demonstrated an increase or improvement (responder vs. non-responder). Seven participants ranked their difficulty related to individualized social media goals, while two had missing data. Descriptive statistics and a Wilcoxon Signed-Rank test was used to analyze the change in goal difficulty following completion of the SELFI program.

## Results

### Primary Analysis: Feasibility and Acceptability

#### Enrollment and Randomization

**Fig. 1:**
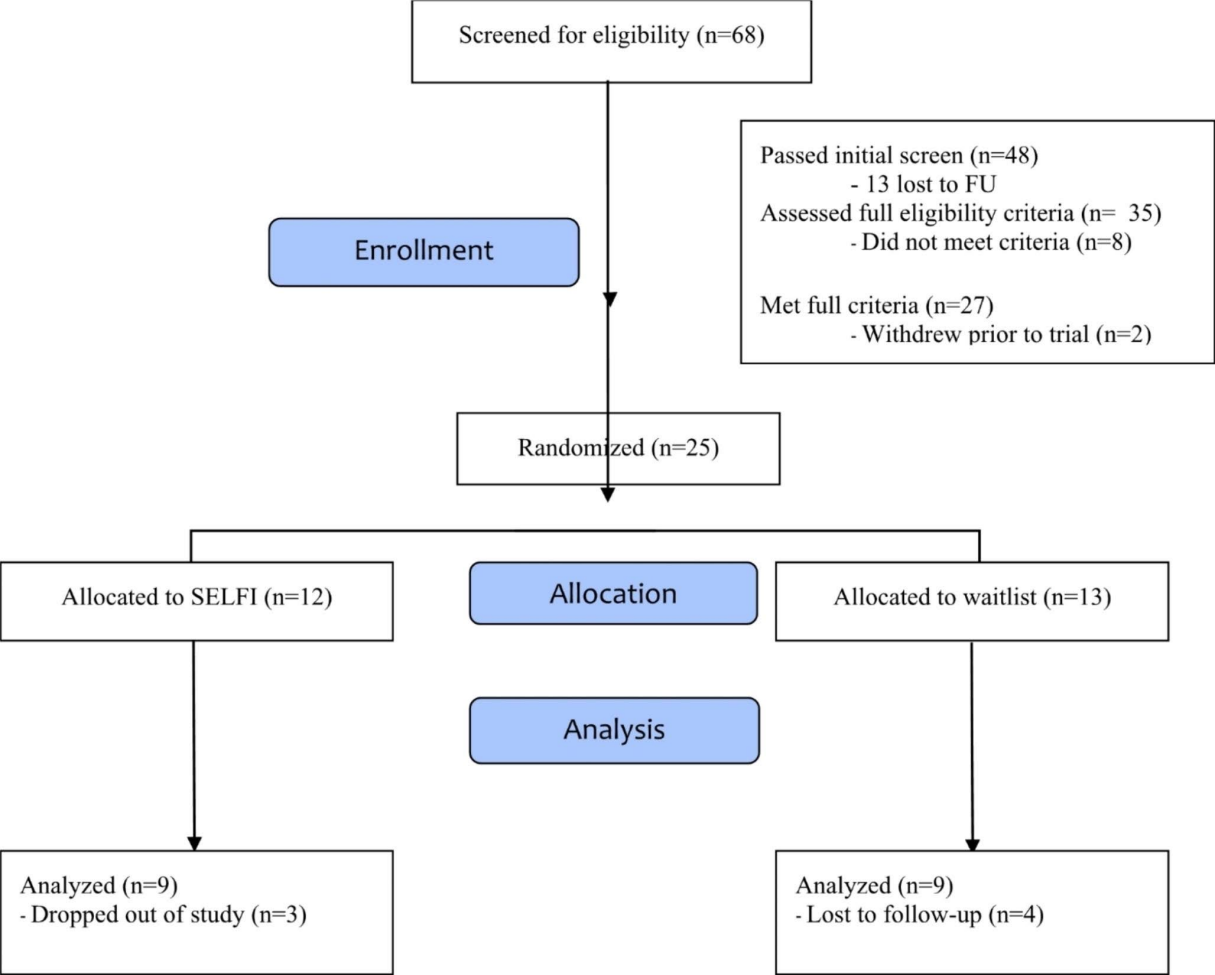
CONSORT flow diagram

A total of 68 individuals completed the online screener within 30 days of recruitment. Of those screened, 48 met initial criteria and 20 were excluded for the following reasons: no Facebook account (10), aged over 40 years (three), aged under 18 years (three), not in the United States (one), and not having an autism diagnosis (three). Of the 48 who passed the initial screen, 13 could not be reached for follow-up after three attempts to schedule an intake appointment. Thirty-five individuals were assessed for full eligibility. Eight were ineligible due to verbal composite scores that fell below the cutoff. In total, 27 individuals met full inclusion criteria, however two withdrew prior to randomization due to scheduling conflict between work and the established program schedule. Twenty-five participants enrolled in the trial and were randomized. There were no significant differences between conditions for age, KBIT-2 verbal composite, and SRS-2 total t-scores, suggesting equivalency. See Fig. 1 for an enrollment summary.

#### Attrition and Attendance

Seven of the enrolled 25 participants (28%) were lost to attrition. Three of the 12 participants assigned to the experimental condition dropped from the trial. One discontinued because of a variable work schedule and one due to feeling uncomfortable in group settings. One participant in the experimental group attended the first eight sessions but did not attend the graduation at week 9 and could not be reached to complete the study. Four participants assigned to the control condition were lost to follow-up. All nine waitlist participants who enrolled in the SELFI program completed the intervention. The mean attendance rate for all 21 participants who enrolled in the SELFI program was 82.8%. The average number of attended sessions per participant was 7.5.

#### Fidelity of Delivery

Adherence to treatment fidelity checklists was at 100% for reviewed recordings. All intended program objectives were completed within this trial.

#### Participant Feedback

Participants who completed the SELFI program reported a high level of agreement regarding treatment satisfaction (*M =* 4.3; SD = 1.1) and enjoyment (*M =* 4.4; SD = 1.1). Most agreed that the program helped them learn new social media skills (*M =* 4.5; SD = 1.1), increased their level of confidence online (*M =* 4.4; SD = 1.1), and supported their individualized social media goals (*M =* 4.3; SD = 1.1). There was moderate agreement regarding use of SELFI skills (*M =* 3.9; SD = 1.1), feeling safer online (*M =* 3.9; SD = 1.1), making new friends (*M =* 3.8; SD = 1.2), using social media to maintain current relationships (*M =* 3.8; SD = 1.1), being more active online (*M =* 3.8; SD = 1.1), and comfort with navigating online romance (*M =* 3.7; SD = 1.1).

The most helpful aspects of the program included use of visual support (e.g. presentation slides, video examples, and handouts), group discussions, practice opportunities, peer mentorship, socializing with others, and learning more about social media features. One participant noted that sharing slides during sessions “provided a visual of the different things that we were learning.” Another participant specified that the most helpful lesson was the topic on maintaining relationships using social media. Favorite components of the program related to the use of peer mentors, group discussions, socializing with other group members, visual supports, practicing skills within breakout rooms, and the lesson on dating and online romance. Least favorite aspects of the SELFI program included, focusing too much on Facebook, sorting through all of the information at once, sharing within group discussions, and some parts of the curriculum seeming too obvious. Recommendations for future programming included adding support for balancing work-life and social media use and support for other online environments (e.g., small talk within group chats). One participant shared “occasionally I get super casually worded messages with a lot of emojis and things and I don’t exactly know the best response.” Participants shared suggestions for improving the SELFI program, which included: a longer program, longer sessions (“hour and a half would be good”), more videos, and the addition of an anonymous discussion forum where participants could ask questions while limiting social anxiety.

#### Peer Mentor Feedback

Four peer mentors supported the SELFI program and completed a feedback survey. Peer mentors reported enjoying the program (*M =* 4.8; SD = 0.4) and perceiving that participants benefited from their mentorship (*M =* 4.8; SD = 0.4). Mentors noted enjoying use of breakout rooms to provide individualized support. Dislikes included inconsistent attendance from certain mentees and limited interaction during didactic lessons. Mentors highlighted the importance of establishing rapport with their mentee by casually checking-in, inviting mentees to share, and not pushing an agenda onto them. Strategies used to support mentees included sharing personal experiences, summarizing lessons during check-out, comprehension checks, and inviting participants to share their screen during breakout room activities so that they could get feedback. Programmatic suggestions included increased opportunities for participants to share, use of the chat function within sessions, and more support for social media platforms other than Facebook.

### Secondary Analysis: Efficacy Outcomes

#### Social Media Experience Scale

A Hotelling’s T^2^ (one-way MANOVA) was run to determine the effect of condition on SMES utility and anxiety subscales. Preliminary assumption checking revealed that data were normally distributed, as assessed by Shapiro-Wilk test (p = .05), and there were no univariate or multivariate outliers, as assessed by inspection of boxplot. Both conditions experienced positive DS for SMES utility and negative DS for SMES anxiety subscales. DS for SMES-Utility was greater (more utility) for the control condition (M = 1.2, SD = 1.1) than the experimental condition (M = 0.6, SD = 1.1). DS for SMES-Anxiety was more negative (reduced anxiety) for the experimental condition (M = -0.5, SD = 0.7) than the control condition (M = -0.1, SD = 0.7). The differences between conditions on the combined dependent variables was not statistically significant, F(1, 15) = 1.31, p < .91; Wilks’ Λ = 0.99; partial η2 = 0.01.

#### Facebook Engagement and Improvement

At the conclusion of the trial, 44.4% (4/9) assigned to the experimental condition had increased Facebook likes/reactions compared to 11.1% (1/9) in the control group. There was not a statistically significant difference in proportions of 0.33, p = .29. Regarding Facebook comments, 44.4% (4/9) of the experimental group were responders compared to 0% (0/9) in the control condition. There was not a statistically significant difference in proportions of 0.44, p = .08. For Facebook posts, 44.4% (4/9) of the experimental group were responders compared to 0% (0/9) in the control condition. There was not a statistically significant difference in proportions of 0.44, p = .08. Of the participants in the experimental group, 55.5% (5/9) were rated as having improved Facebook engagement compared to 0% (0/9) in the control group. There was a statistically significant difference in proportions of 0.55, p = .03. See Table 3 for a summary of response rates between conditions for Facebook variables.

**Table 3:** Responders vs. non-responders for outcome variables

	SELFI Responders	SELFI Non-Responders	Waitlist Responders	Waitlist Non-Responders	Statistical Significance
Likes/ Reactions	4	5	1	8	0.294
Comments	4	5	0	9	0.08
Posts	4	5	0	9	0.08
Improvement Ratings	5	4	0	9	0.03*

**Note:** Responders demonstrated an increase in frequency or rating between baseline and week 9. Non-responders demonstrated no change or a reduction in frequency or rating between timepoints. Significance for Fisher’s exact tests, reported in p-value. *Statistically significant

### Individual Goals

Of the seven participants who reported data, six rated a decrease in goal difficulty, whereas one saw no change. A Wilcoxon signed-rank test determined that there was a statistically significant median decrease of 3 in goal difficulty from baseline (median rank = 6; “difficult”) to week 9 (median rank = 2; “easy”), z = -2.2, p = .03.

## Discussion

The primary purpose of this study was to pilot test the novel SELFI program with autistic adults for feasibility and acceptability. A secondary aim was to assess signals of preliminary efficacy using a randomized controlled trial. Considering the pilot nature of this program, an emphasis was placed on identifying programmatic strengths and areas for improvement to inform future studies and program development. Findings from this study support the innovative SELFI program as feasible considering data collected from enrollment, program delivery, and protocol execution. Feedback from participants and peer mentors who completed the SELFI program described their experience as acceptable and helpful for developing online confidence and social competence. Despite the sample being underpowered, findings from the trial reflect statistically significant differences in response rate regarding improved social engagement on Facebook and trending increases in the frequency of specific behaviors between the experimental and control conditions. This study serves as an exciting next step in the development of a social media and internet safety program for autistic adults. 

Considering the novelty of this type of study, an important research question related to whether young autistic adults were interested in participating in a targeted social media program. A recruitment goal of 24 participants was chosen in consideration of Julious (2005), who recommends that pilot studies attempt to include at least 12 participants per treatment group to assess differences on continuous variables. The remote nature of this study enabled the recruitment of participants from various geographical regions, which increased access to the study. By connecting with relevant organizations through digital communication (e.g. email, social media), this study solicited a large volume of interest and the recruitment goal was met within one month. While successfully enrolling target participants supports program feasibility, there is room for improvement considering that the enrollment rate (those screened vs. enrolled) reflected significant attrition. Although the interest screener was successful at identifying the target population, only 25 of the 68 screened participants enrolled in the study. Several individuals with diverse presentations (age, verbal ability, etc.) and internet experiences (e.g. social media preferences) expressed interest in the study but were excluded due to strict inclusion criteria. Restricting criteria to the included parameters limits its feasibility and acceptability since many autistic people are currently unable to participate. Since youth with disabilities (Wells & Mitchell, 2014) and older internet users (Hunsaker & Hargittai, 2018) face heightened challenges online, exclusion of these subpopulations limits accessibility to those in high need. To improve the feasibility and acceptability of the SELFI program, participation in future studies should have more inclusive criteria.

Another important area of exploration was the acceptability of the SELFI program when delivered virtually in group format. The initial prototype of the SELFI program reflected preliminary acceptability (REDACTED FOR ANONYMINITY et al., under review), however changes to program format required additional research. From a practical perspective, delivering the SELFI program over telehealth was preferable due to the COVID-19 pandemic which limited in-person interaction. Clinically, adapting the curriculum for group format was intended to increase access (Sutherland et al., 2018) and align SELFI with established evidence-based programs for face-to-face socialization (Spain & Blainey, 2015). As reflected by consistent attendance rates and limited attrition, participants were capable and motivated to consistently participate in the SELFI program and had flexibility to overcome access constraints. By delivering SELFI using telehealth, participation was not limited to geographical location within the United States, allowing participants to join from various circumstances (e.g. in their car, after work). Participants reported that group discussions and socializing with others were favorite aspects of the intervention, indicating that group work is a strength of the program. However, a few participants noted feeling uncomfortable sharing aloud within group discussions and therefore future programs should explore use of augmentative and alternative communication (AAC) and other forms of non-verbal participation. While group format is a critical aspect of the SELFI program, implementation is not necessarily restricted to telehealth. Although more testing is needed, delivery of the piloted protocols could theoretically be executed while face-to-face, though minor adaptations may be necessary (e.g. showing videos on a projector vs. screen share). Related to feasibly executing the program protocol, the current SELFI program was delivered with 100% fidelity across all reviewed sessions. All intended objectives were achieved, in part, due to the curriculum being outlined and shared on the screen during sessions. In addition to ensuring that all material was delivered with consistency, use of visual support was intended to support participants with structure, predictability, and comprehension (Krasny et al., 2003). Participants noted that visual support such as powerpoint slides, videos, and handouts, were preferred elements of the program and therefore should be prioritized within future social skills programs. If adapted to in-person implementation, program delivery should explore ways to integrate visual cues, including use of pictograms.

Feedback from participants and peer mentors provided valuable information regarding the acceptability of the piloted SELFI program. The program was noted to be enjoyable and satisfactory, and most participants reported learning new skills and feeling more confident online. These findings are consistent with feedback from peer mentors who prioritized a casual and fun intervention environment, promoted a strengths-based approach, and supported self-efficacy. While enjoyment, increasing knowledge, and improving confidence are important elements of the piloted program, there are areas of needed improvement in order to increase the acceptability of the SELFI program. The intention of the SELFI program is to support safe and effective online interactions, however participants reported lower levels of agreement with several questions related to socially engaging with peers online. As it’s currently developed, the SELFI program is less acceptable for improving one’s sense of internet safety, being more active online, and using social media to maintain and develop social and romantic relationships. In order to better align SELFI with its intended objectives, future program development should identify additional content and strategies to better support participants with internet safety and leveraging the internet to improve desired relationships. Recognizing that autistic people may have varying levels of experience and interests regarding social media (Zhao et al., 2019), future studies should expand the scope of sessions to include skills related to broader internet use and computer-mediated communication, including platforms other than Facebook. Possible additions to the curriculum could include being concise within text messages and participating in group chats.

A secondary aim of the study was to explore the preliminary efficacy of the SELFI program. Considering the pilot nature of this program and the underpowered sample size, an emphasis was placed on identifying clinically relevant changes in social media engagement. Comparison of Facebook engagement before and after the study indicated that about half (4/9) of the participants who completed the SELFI program saw increased engagement within all measured socially relevant Facebook variables (likes/reactions, comments, and posts). No participants assigned to the waitlist condition observed increased social engagement on all Facebook variables. Considering that more social interactions with peers can improve social competence (Vernon et al., 2012), improving the frequency of engagement on social media may benefit the development of competency related to navigating online socialization. This being said, while a higher frequency of social media activity is indicative of increased social participation, being too active online and oversharing can have negative consequences on relationships (Pham et al., 2019). While these findings highlight the number of participants that demonstrated increased social media engagement, using the frequency of Facebook engagement as a barometer of improved social competence is imperfect. For example, one SELFI participant increased their likes/reactions from zero at baseline to 1300 at week 9, which may reflect overactivity. Within the current context, there is incomplete information regarding the relationship between increased Facebook activity, online social engagement, and social consequences since the impact of specific behaviors are context dependent.

Recognizing the understudied nature of social media skills and competency, an important area of exploration was identifying appropriate outcomes that would capture the hypothesized mechanism of change. Frankly, little is known about assessing changes in social media behavior and this study served as an important step in piloting approaches to measurement. Common measures of social engagement within face-to-face interactions include assessing the frequency of social interactions, engagement within conversations, and survey measures assessing knowledge related to social interaction (Estabillo et al., 2022; Vernon et al., 2016). While these outcomes provide meaningful insight into behavior relevant to in-person interactions, there is limited utility for understanding online socialization. As mentioned above, the frequency of online interaction provides meaningful but incomplete information and therefore a creative approach was piloted. In an attempt to identify clinically meaningful changes in online social competency, this study sought to assess overall improvements in Facebook usage. While perceived improvement on social media is a relatively broad construct, the purpose of this variable was to attempt to capture an outcome sensitive to change and inclusive of the frequency and substance of interactions. This being said, significant effort went into developing a measure that is inclusive of autistic people and non-judgmental of neurodiverse preferences. Recognizing potential neurotypical bias when interpreting autistic behavior and the need to make autism research better aligned with the neurodiversity movement (Schuck et al., 2021), an emphasis was placed on including both autistic and non-autistic research assistants to serve as raters to provide diverse perspectives. To control for gender differences in online socialization (Zheng et al., 2016), an even balance of male and female perspectives were gathered. Acknowledging that complex intersecting factors influence perception of behavior improvement, the research team developed a protocol to quantify improvement for each participant. This protocol included presenting screen shots of participant behavior at baseline and after the trial and asking raters to assess level of improvement considering frequency and the content included within interactions. Each participant’s overall Facebook improvement score represents the average score of 12 similarly-aged male, female, autistic, and non-autistic peers. The innovative protocol is inclusive of diverse perspectives and its social validity should be considered a strength, though more research is needed to assess its construct and criterion validity.

Findings from these ratings highlight that a majority (5/9) of participants in the experimental condition were rated by autistic and non-autistic peers to have improved Facebook engagement while none were improved in the control condition. Unique about these findings is that participants with an increased frequency of Facebook engagement were perceived to have improved online engagement, in addition to one participant who had no increase in frequency. This implies that being more socially active on social media may help improve friendship quality, however there may be other factors that influence social outcomes aside from frequency. To develop relationships online, it takes more than just having a social media account – one must actively post and engage with others in order to form connections with others. By increasing social engagement with peers, participants may have more opportunities to develop and maintain social relationships, which may lead to more positive social outcomes (Vernon et al., 2012). While increasing social engagement could be valuable, developing and maintaining relationships is important and should be regarded as important outcomes. In addition to being active, safe and successful internet use requires awareness of how to best navigate interpersonal interactions and the social consequences of their online behavior. These findings imply that compared to a control condition, participation in the SELFI program may help improve online social competency, though more research is needed, including the collection of follow-up data.

Related to clinical outcomes, there were no significant differences between groups for social media utility and anxiety. This being said, both groups demonstrated a reduction of social anxiety symptoms and reported feeling more positive about using social media. The timing of this study and historical artifacts likely impacted these findings. The trial took place during the COVID-19 pandemic, starting in February and ending in April 2021, which was an especially turbulent period for social anxiety and internet use. During this time, autistic adults experienced a range of mental health challenges, compounded by changes in the social world, living with uncertainty, disruptions to self-regulation, and barriers to basic needs (Bundy et al., 2022). Many autistic adults experienced heightened anxiety during the pandemic (Mosquera et al., 2021), which may have influenced change scores during this trial. Additionally, limited in-person interaction increased society’s dependence on social media for socialization, likely influencing increased utility scores for both conditions. The confounding impact of these historical artifacts makes the impact of SELFI on clinical outcomes difficult to ascertain. Noteworthy about the data is that participants enrolled in the SELFI program demonstrated a greater (non-significant) reduction in social media related anxiety than the control group (-0.5 vs. -0.1), which aligns with the hypothesized mechanism of change. These data reflect signals of promise, though future studies with a larger sample size are necessary to provide further information regarding program efficacy.

### Limitations and Future Directions

The present study is promising, however there are several limitations. The first is that all participants identified with binary genders and most were male. Although an approximate ratio of 4:1 should be expected for samples of autistic individuals (Loomes et al., 2017), females and non-gender conforming people often have unique approaches and needs regarding online socialization (Zheng et al., 2016). Considering that females are more likely than their male counterparts to experience riskier online interactions (Padilla-Walker et al., 2010), incidences of cyberbullying (Wang et al. 2020), cyberstalking (Reyns et al., 2018) and unwanted sexual solicitation (Baumgartner & Morris, 2010), additional support may be needed for female internet users. Additionally, since autistic individuals are more likely to identify as trans or non-binary (Warrier et al., 2020), it is important that social support for autistic people include gender diverse samples and consider unique gendered needs regarding socialization.

Another limitation of this study was the exclusion of autistic adults with lower verbal abilities so that participants could be grouped by language level (Krasny et al., 2003). The status quo of clinical research has been to use standardized assessments (e.g. cognitive test) to characterize participants and to control for confounding variables. Social skills programs have traditionally excluded individuals with limited spoken language and those with intellectual and developmental disabilities (IDD), however recent studies have prioritized supporting this vulnerable population (Ferguson et al., 2021). While verbal ability may be a relevant proxy for participation in certain programs, online interaction changes the requirements for verbal engagement. Since technology such as augmentative and alternative communication (AAC) devices have increased access for autistic people (Donaldson et al., 2021), excluding those with differing verbal abilities may be unnecessarily inequitable. Future studies related to social media should explore adaptations to protocols to be inclusive of autistic people of all presentations.

This study is also limited by focusing too much on Facebook. The SELFI program is intended to target generalizable social media skills, however much of the available literature has focused on Facebook, which has traditionally been the most popular (Ward et al., 2018). In addition to its popularity, Facebook was a focus of this study due to being the only major social media platform to offer a standardized approach to accessing a chronological log of online behavior via the DYI feature. Within the context of Facebook, participants in the experimental condition demonstrated increased and improved activity compared to the control condition, however all participants were aware that Facebook behavior would be evaluated within the trial, which limits the validity of these findings. Considering that about 70% of young adults report being active on at least three social media sites (Primack et al., 2017), it is possible that these data provided only a small snapshot of social media activity. As the program is currently constructed, focusing too much on Facebook may threaten the face validity of a generalized social media skills program. Refinement to the SELFI program should allow participants to choose specific target social media platforms and adapt the contents of the program to match participant interests. Additional study limitations include not examining adverse events (e.g., cyber-victimization) related to increased social media use and individual goals not using a validated goal scale. To better inform the generalizability of research findings, future studies should include more established outcomes (e.g., quality of life), goal scaling (e.g., Goal Attainment Scale; Kiresuk et al., 2014), and examine outcomes related to adverse experiences online. Considering that increased online social engagement may increase vulnerability to the negative effects of social media use, future iterations of the SELFI program will prioritize more support for internet safety and assess outcomes related to the negative consequences of internet use.

The biggest limitation of this study is that the SELFI program was developed without input from stakeholders in the autism community. Support programs for autistic individuals have traditionally excluded their perspectives and as a result, have led to unintended negative consequences (Chapman & Bovell, 2022). Although the SELFI program was conceived in consideration of autistic adults who sought clinical support with social media use, autistic individuals were never consulted regarding program or study procedures. The basis of this research assumes that autistic individuals would benefit from more social interaction online, considering previous research connecting loneliness, friendship, anxiety, and social media use (Mazurek, 2013, 2014; van Schalkwyk et al., 2017; Ward et al., 2018). While this study’s approach was informed by available literature, the SELFI program could be strengthened by engaging various stakeholders within the autism community to inform the overarching aims of the program itself. For the SELFI program to be more inclusive of neurodiversity, future studies must collaborate with and integrate the needs and perspectives of autistic individuals with varying needs and perspectives. Strategies to improve program practices include centering autistic voices, supporting an autistic “way of being,” assessing social and ecological validity, prioritizing participatory research, and using adaptive intervention designs (Schuck et al., 2021). Collaboration with autistic people with diverse presentations (e.g. age, gender, abilities), caregivers, and providers of autism services is needed in order to ensure that the SELFI program is inclusive and consistent with stakeholder needs. Future research includes collaboratively and iteratively redesigning the SELFI program to enhance its usability prior to more rigorous testing.

## Conclusion

We live in an increasingly digital world that makes computer-mediated communication and online socialization more accessible to autistic people. The SELFI program represents one of the first social media skills programs that is committed to supporting safe and effective access to social media and the internet. This study establishes a promising foundation regarding the feasibility and acceptability of the SELFI program for autistic adults, however more research and development is needed in order to make it more inclusive and usable. As the it is currently constructed, the SELFI program may be helpful for supporting autistic adults without intellectual disability with increasing online social engagement and improving upon their individualized social media goals. However, the SELFI program may not be appropriate for individuals with higher levels of needed support, including those with IDD, and adaptions may be needed to make the intervention more inclusive. These findings establish an exciting first step forward in the understudied area of supporting safe and effective social media use amongst autistic people, although further refinement is needed prior to engaging in a larger trial. Future directions include collaborating with the autism community to enhance the usability of the SELFI program, refining the program to better support diverse needs and presentations, and testing the SELFI program using more rigorous research methodologies.
